# Study of Oncolytic Virus Preservation and Formulation

**DOI:** 10.3390/ph16060843

**Published:** 2023-06-05

**Authors:** Lina Pan, Xiyu Liu, Dianfa Fan, Zhangbo Qian, Xinjun Sun, Pan Wu, Liping Zhong

**Affiliations:** 1 State Key Laboratory of Targeting Oncology, National Center for International Research of Bio-Targeting Theranostics, Guangxi Key Laboratory of Bio-Targeting Theranostics, Collaborative Innovation Center for Targeting Tumor Diagnosis and Therapy, Guangxi Medical University, Nanning 530021, China; 15777202033@163.com (L.P.); liuxiyu419@163.com (X.L.); fandf1996@163.com (D.F.); qianzhangb@163.com (Z.Q.); m17854337107@163.com (X.S.); 2 School of Pharmacy, Guangxi Medical University, Nanning 530021, China

**Keywords:** oncolytic virus formulation, formulation design, excipients, degradation mechanism

## Abstract

In recent years, oncolytic viruses (OVs) have emerged as an effective means of treating cancer. OVs have multiple oncotherapeutic functions including specifically infecting and lysing tumor cells, initiating immune cell death, attacking and destroying tumor angiogenesis and triggering a broad bystander effect. Oncolytic viruses have been used in clinical trials and clinical treatment as drugs for cancer therapy, and as a result, oncolytic viruses are required to have long-term storage stability for clinical use. In the clinical application of oncolytic viruses, formulation design plays a decisive role in the stability of the virus. Therefore, this paper reviews the degradation factors and their degradation mechanisms (pH, thermal stress, freeze–thaw damage, surface adsorption, oxidation, etc.) faced by oncolytic viruses during storage, and it discusses how to rationally add excipients for the degradation mechanisms to achieve the purpose of maintaining the long-term stability of oncolytic viral activity. Finally, the formulation strategies for the long-term formulation stability of oncolytic viruses are discussed in terms of buffers, permeation agents, cryoprotectants, surfactants, free radical scavengers, and bulking agent based on virus degradation mechanisms.

## 1. Introduction

OV therapy has vast applications in the field of oncology treatment for cancer. A number of viruses have been investigated as oncolytic viruses and have proceeded to the clinical trial stage. Clinical studies have demonstrated that OVs have multiple therapeutic functions: First, OVs have been shown to trigger systemic anti-tumor immunity in animal models and patients [1,2]. Second, bystander killing can be triggered in different ways by inserting therapeutic genes [3]. Finally, OVs have been shown to induce endothelial cell death in tumor vessels, leading to the vascular closure and indirect destruction of tumor cells [4]. Obviously, the quantitative clinical application of oncolytic viruses is very important for clinical data collection as well as for quantifying treatment effects. The long-term formulation stability of viruses is influenced by several factors. The first involves the issue of thermal stress. Most oncolytic virus formulations, and even attenuated or inactivated vaccines of the virus, are stored only at ultra-low temperatures or in freeze-dried form at low temperatures; for example, the FDA-approved T-VEC requires storage at −90 °C to −70 °C [5] and Merck’s measles vaccine Attenuvax^®^ is commercially available as a lyophilized formulation [6]. Viruses are exposed to a variety of other degradation risks when preserved including freeze–thaw damage, pH changes, surface adsorption, shear stress, and oxidative denaturation. For example, the crystallization of buffers during the freezing process can lead to pH changes, especially in sodium phosphate buffers [7], where both overly high and overly low pH can cause virus denaturation [8,9]. In addition, low pH values cause viral aggregation, which may cause irreversible changes by viral denaturation [10]. Consequently, choosing the right pH is one of the key factors in achieving long-term preservation. In addition, surface adsorption is also one of the main factors affecting the long-term preservation of viruses. Firstly, the virus can adsorb onto drug containers. Secondly, crystallization during the freezing crystallization process can lead to surface induced aggregation, resulting in significant losses. In one study, the use of a 1 mL syringe resulted in a 75% loss of virus [11]. Oxidation can be caused by a variety of factors including excipients that maintain virus stability such as buffers, surface adsorbents, sugars, and salts; dissolved oxygen, light, and amino acids that are susceptible to oxidation can all be factors in oxidation. Negligence in any of these areas during virus preservation can lead to a significant loss of viral activity. A 1A rational formulation design is required to address these challenges in virus preservation. This includes the use of appropriate buffers, cryoprotectants, surface adsorbents, osmolytes, antioxidants, and bulking agents.

## 2. Types of Oncolytic Virus

Hundreds of oncolytic virus programs are currently in clinical trials [12], and several oncolytic viruses have been approved for marketing. T-VEC [13], a second-generation herpes simplex virus type 1 (HSV-1) with recombinant granulocyte macrophage colony-stimulating factor (GM-CSF), was approved as the first oncolytic virus in the United States and Europe [14]. Additional oncolytic viruses approaching drug approval in North America and Europe include cowpox virus JX-594 (pexastimogene devacirepvec) for hepatocellular carcinoma [15] and adenovirus CG0070 recombinant GM-CSF for the treatment of bladder cancer [16]. In Japan, a phase II clinical trial of the third-generation oncolytic virus HSV-1 G47Δ [17] in patients with glioblastoma is underway. In general, the more widely studied OVs include adenovirus, herpes simplex virus, Newcastle disease virus, measles virus, bean mosaic virus, and coxsackievirus.

## 3. Viral Degradation Factors

Before developing a formulation strategy, we first needed to understand the factors that lead to viral degradation during the storage of viral solutions and their degradation mechanisms. The stability of viruses is closely related to the conformation of viral proteins. It might be clearer to specify that “the stability of viruses is closely related to the conformation of their capsid proteins, which significantly affects viral stability during storage [18]. Therefore, some theories related to protein stability have been cited in this paper. Factors that can affect the long-term formulation stability of viral agents are typically categorized as physical or chemical factors; that being said, it should be noted that some factors may have aspects of both categories.

## 4. Chemical Factors

### 4.1. pH

pH is one of the important factors that determines the stability of viruses. Both excessively high and excessively low pH levels can lead to a loss of viral activity. Viruses aggregate at low pH and at high pH levels, and viruses form empty capsids [10]. An unsuitable pH level can cause the coat protein to change from a folded to an unfolded state, and as a result, the Tm (melting temperature) of the virus is affected by changes in pH [19]. For example, adenovirus formulations are very sensitive to pH changes when frozen; it has been shown that formulations maintained at the initial pH (7.4) showed no significant titer loss after freezing and thawing, whereas viral formulations that underwent a 3 pH unit drop without freezing lost 1 log of titer, and frozen formulations that underwent a 3 pH unit drop lost 0.5 log of titer [20].

Therefore, researchers have begun to study how pH affects viral stability. As the solution pH approaches the viral isoelectric point (PI), hydration around the virus is reduced, leading to further irreversible denaturation of viral aggregates [21]. Moreover, the closer the pH is to the isoelectric point, the lower the solubility of the protein. The n-terminal portion of the measles virus phosphoprotein has been used as a model for disorder. The proteins tested showed lower solubility close to their PI. The overall solubility of the protein can be determined by the region of the protein that has a higher net charge per residue [22], with a lower net charge closer to the isoelectric point. This may lead to the aggregation of virus precipitation at high viral concentrations. Viruses are typically only stable within a narrow pH range, as the pH level affects the net charge on the protein molecule and the nature of electrostatic interactions [9,23]. In general, the higher the net charge, the lower the tendency to aggregate due to electrostatic repulsion and the higher the colloidal stability [24].

The pH level affects the net surface charge of viruses, which in turn, affects their thermal stability. In an experiment found that the Tm of the measles virus varies with changes in pH. The Tm of samples with pH 6 and 7 is close to 50 °C, while the Tm of samples with pH 5 or 8 is slightly lower, approximately 47 °C [25].

The physical stability of the viral capsid generally increases as the pH decreases (pH 8–5), and a pH less than 5 generally leads to significant aggregation of the virus and consequent loss of viral activity (Table 1). For example, REXROAD et al. investigated the thermal stability of adenovirus type 5 (Ad5) at pH 3–8 using various biophysical techniques at both high and low ionic strengths. They found that the capsid stability of Ad5 increased with decreasing pH at both ionic strengths, while at low ionic strengths, significant aggregation was observed at a pH less than 5 [9]. Although the nuclear capsid can maintain higher stability under acidic conditions, many studies have suggested that weak acidic conditions trigger a viral decapsidation effect, causing remodeling of the capsid protein. Some of the oncolytic viruses that have been shown to decapsidize under weak acidic conditions are Newcastle disease virus [26], adenovirus [27], herpes simplex virus [28], and coxsackievirus [29]. For example, weak acidic conditions cause the nucleic acids of adenoviruses to coalesce, and stronger acidic conditions are observed at pH 4 when the capsid is stiff and more prone to rupture, while structural rearrangement of the capsid protein can be observed at pH 6 [27]. Dissociation of adenovirus from the capsid apex shows PH-dependent capsid breakdown [23]. Ad5 viral particles are susceptible to violent dissociation when overnight acidification at 4 °C affects particle morphology and genome accessibility [27]. Viruses were also found to maintain better activity at pH 7.2–7.4 in adenovirus formulation studies [30]. Thus, due to conflicting results regarding the effect of acidic pH on capsid stability, further studies using different conditions and techniques are necessary to better understand this phenomenon.

### 4.2. Surface Adsorption

**Figure 1:**
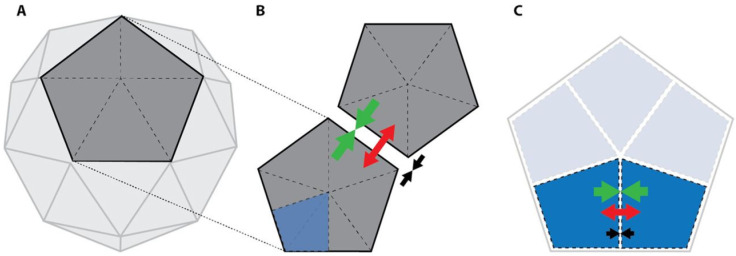
Structural features and interaction forces of the adenovirus virus shell. Green, van der Waals forces; red, electrostatic force; black, overall interaction force. (**A**) Schematic diagram of the icosahedron capsid structure. (**B**) Schematic diagram of the pentamer interaction force. (**C**) Schematic diagram of atomic interaction forces. Reproduced from reference [40] with no copyright restrictions.

Adsorption is one of the main causes of virus titer loss during storage. For instance, when adenovirus is stored at 30 °C for 2 weeks without Tween 80 in the formulation, there can be a loss of infectious titer by more than 2 logs [36]. Armanious et al., calculated the isoelectric point of viruses based on ionizable amino acids located on the external surface of their capsids. They also studied the adsorption of these viruses on surfaces under various pH conditions and found that the surface activity of viral particles is primarily influenced by long-range electrostatic and hydrophobic interactions. In contrast, short-range van der Waals interactions, hydrogen bonding, and spatial effects had a lesser impact. [37]. In turn, the viral charge is related to the pH and ionic strength. First, Guillaume Bastin et al. measured the variation in MS2, GA, and Q phage with a pH from 4 to 11 by CZE analysis and found that viral hydrophobicity varies with pH; the closer to the isoelectric point the stronger the hydrophobicity [38]. Secondly, when the viral surface is more highly charged, the viruses already adsorbed on the solid surface prevent the accumulation of more viruses to the surface through electrostatic interactions. Furthermore, as the viral charge increases, the exposure of viral hydrophobic proteins decreases. The increased ionic strength shields the double layer and thus the electrostatic force, leading to the attachment of viruses to the surface through interactions (e.g., hydrophobic effects) [39]. Therefore, selecting a suitable pH and mitigating ionic strength can reduce the surface adsorption of viruses. However, Simon Meister et al. found that the stability of the viral capsid arises from the van der Waals forces of attraction between the proteins inside the viral capsid (Figure 1). An increase in ionic strength resulted in a significant increase in van der Waals attraction at the interface. They determined the thermal stability of four enterovirus strains in solutions with different pH values and different NaCl concentrations. Through molecular simulation of protein interactions at the interface between virus capsid pentamers, combined with experimental results, it was found that an increase in NaCl concentration resulted in a 20 °C increase in the fracture point temperature of all tested viruses [40]. Therefore, overly high ion concentrations can lead to surface adsorption, while overly low ion concentrations can reduce nucleocapsid stability. We need to further determine the concentration when adding ions, both to facilitate the addition of ions to maintain the stability of the capsid and to not affect the charged nature of the virus caused by the ionic strength being too high, leading to increased exposure of the hydrophobic side chain of the virus causing more surface adsorption and aggregation of the virus. Again, because each virus has a different charge, the concentration of added ions may be different for different viruses.

### 4.3. Thermal Stress

The transition temperature (Tm) and Gibbs free energy associated with the stability of viral capsid proteins are small, so that slight temperature increases may lead to viral aggregation and protein denaturation. The difference in free energy between natural (biologically active) and unfolded (inactive) protein conformations may be very low (~5 to 20 kcal/mol) [41]. Storage of viruses at ambient temperatures may lead to the rapid unfolding of epitopes, loss of capsid integrity, reorganization of major viral conformations, and loss of key protein functions, resulting in the immediate loss of viral activity [42]. Currently marketed oncolytic virus and vaccine formulations are usually frozen at ultra-low temperatures or made into lyophilized formulations and stored at low temperatures. The long-term storage of viruses at room temperature or 2 to 8 °C is a problem that needs to be overcome to allow the widespread use of viral formulations. Pelliccia et al. increased the half-life of adenovirus expressing green fluorescent protein from 48 h at 37 °C to 21 days (from 7 to 430 days) at room temperature by adding anionic gold nanoparticles or polyethene glycol. They replicated the known stabilizing effect of sucrose, but at concentrations that were several orders of magnitude lower [43]. They then proposed that surface modification of the protein could significantly increase the Tm value. Their modification involved coupling N,N’-dimethyl-1,3-propanediamine onto the globular protein avidin and cationizing the protein solution. This was followed by complexing it with the anionic surfactant glycolic acid ethoxylated lauryl ether to produce a solvent-free biofluid that further increased the temperature Tm of the protein from 74.3 °C to 139.0 °C at 10 °C (an increase of 71.7 °C compared to the protein in aqueous solution) [44]. Although there have been some studies on preserving liquid formulations at room temperature, the results still require improvement for most viruses. Short-term room temperature preservation stability is the goal of current formulations to accommodate transient temperature changes of viruses during transfer. Long-term storage stability at room temperature requires more in-depth study.

### 4.4. Oxidation and Other Factors

Viral oxidation can be caused by a variety of factors including buffers, polysorbate (PS) [45], reducing sugars, salts, peroxides, metals [36], dissolved oxygen, light [46], and amino acid sequences that are susceptible to oxidation. These factors can act individually or as cofactors for oxidation. Excipients usually contain trace amounts of metals [47]. In addition, metal ions are important factors in the catalytic production of oxidants [36]. Glucose and lactose should be avoided because they are reducing excipients. Dissolved oxygen and light promote amino acid cleavage to produce free radicals. In addition to the above major factors, some other factors also require our attention. For example, agitation can lead to protein unfolding [48]. Overly high viral concentrations can induce aggregation and lead to structural changes [49]. Isomerization is also one of the common chemical denaturation methods of proteins. The most common isomerization in proteins is the formation of aspartic acid, which results from hydrolytic isomerization of aspartic acid through a succinimide intermediate [11].

## 5. Physical Factors

### Freeze–Thaw Damage

In the production of biopharmaceuticals, freezing operations are used to ensure the stability of biologics for long-term preservation, but they also introduce additional stress. It was reported by Evans et al. that an experimental assay with a tissue culture infective dose (TCID50) found that AD5 lost 2 logs of infectivity after a single freeze-thaw cycle in the absence of cryoprotectants [36]. Freeze–thaw cycles cause significant viral aggregation and increase envelope permeability and even nucleocapsid cleavage. The effect of freeze-thaw cycles on the herpes simplex virus has been examined using assays such as MFI and PI fluorescence, which have found a significant increase in the concentration of subvisible particles and an increase in PI fluorescence intensity after one freeze-thaw (F/T) week of the viral formulation [19]. Subsequently, a logistic (sigmoid) function was used to fit the change in volume of the adenovirus capsid with time after freezing-thawing. The first-order derivative of the fitted sigmoid was then calculated, and it was found that the coat of wild-type adenovirus decomposed at a rate of 3 × 104 nm^3^ per minute after freezing–thawing [50]. This led to the study of how freezing affects the stability of proteins or viruses.

Freezing is defined as the process of ice crystallization in supercooled water. The freezing process first involves cooling the solution, then forming a state where the solution is supercooled until ice nucleation occurs [51]. The crystallization of water is first experienced when the temperature reaches the crystallization temperature of the water, Tg (w). Due to the crystallization of water, the solute is rapidly concentrated and consequently causes uneven solute distribution. Seifert et al., evaluated different formulation systems using linear regression of the heat of melting from the beginning of the concentration sequence from Tg to the end of the melting event. They measured the water content of frozen concentrates as 20–30% [52]. At this point, the solute was highly concentrated, and the pH was drastically altered by the crystallization of the buffer components [53,54]. We have previously discussed the effect of pH on viral activity, see the ‘pH’ section of the article ‘Chemical Factors’ Using phosphate buffer as an example, the crystallization of buffer components in solution has been identified using low-temperature X-ray diffractometry. It was first observed that the crystallization of dodecahydrate resulted in a decrease in pH from 7.0 to 4.1 [7]. Thus, preservation below the eutectic glass transition temperature favors the stability of viral or protein formulations. The crystallization stops and forms a glassy state as the eutectic solute temperature is reached with decreasing temperature during freezing. It has been shown that wild-type adenovirus has significantly lower infectious titers after 12 months of storage above the glass transition temperature [55]. Preservation above the glass transition temperature corresponds to the virus being in an environment with drastic pH changes and highly concentrated solutes [52,56].

**Figure 2:**
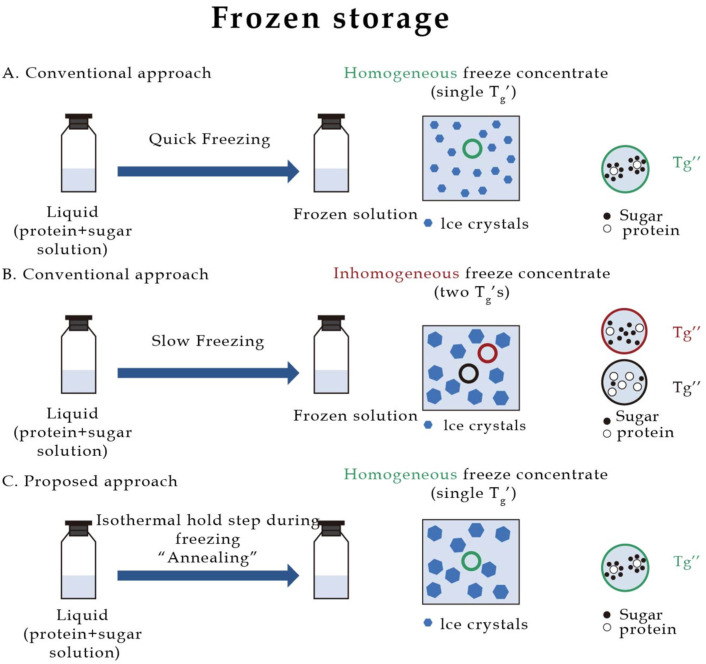
(**A**). Rapid freezing leads to the formation of small ice crystals that increase the surface area of ice water. (**B**). Freezing without an annealing step (held at −20 °C for a certain time) leads to multiple glass transition temperatures (Tg) and inhomogeneous distribution of proteins and sucrose. Sucrose remains amorphous in the frozen state. (**C**). Adding an annealing step to the freezing process produces a single glass ring transition temperature and uniform distribution of protein and sucrose. Data were obtained from [56,58,59].

Subsequently, the rate of cooling was found to be one of the key factors affecting the stability of frozen or lyophilized formulations. Zhai et al. compared the effect of three different cooling rates on the retention of viral infectivity after freezing herpes simplex virus. The freezing conditions studied included frozen metal plate cooling (223 K), nitrogen (SN2), and molten propane (LN2) cooled by injection of liquid nitrogen. SEM showed rapid freezing to form smaller ice crystals. The fastest freezing rate had the lowest recovery rate for LN2, the highest recovery rate for liquid nitrogen, and the largest ice crystals formed from the metal-cooled plates. However, the recovery rate was lower than liquid nitrogen freezing [57]. Larger nuclei were formed during slow freezing with the smaller surface area of ice and water contact. During fast freezing, smaller and more ice crystals were formed during the crystallization of the solution, and the specific surface area (SSA) increased. Hauptmann et al., used 0 °C/min, 1 °C/min, 2 °C/min, and 5 °C/min freezing rates, and slow freezing was observed by the formation of larger ice crystals on OCM images [58]. With fast freezing, the freezing rate increased the ice front velocity, leading to enhanced dendritic ice growth and more efficient ice trapping. The solute was then removed from the ice front by diffusion and convection, leading to a more uniform solute concentration distribution [59]. Minatovicz et al. first measured ice SSA in dry samples of large-volume protein solutions using volumetric nitrogen adsorption isotherms, and the ice surface area increased as the freezing rate increased. After rapid freezing, proteins were more uniformly distributed throughout the frozen solution [60]. However, it is still controversial as to whether the high ice-water surface area due to fast freezing or the uneven solute distribution due to slow freezing determines the stability of biologics. Furthermore, to address the problem of uneven solute distribution during slow freezing, Sonje et al. reported that adding an annealing step to the freezing step can solve the problem of solute concentration. The authors cooled the mannitol-alginate 3:1 samples to −20 °C at a rate of 0.5 °C/min, held them for 2 h, and then further cooled them to −60 °C at a rate of 5 °C/min. Compared to the unannealed control group, it was found that the annealed group had a single glass transition and a uniform distribution of solutes. In contrast, the unannealed group had multiple glass transition temperatures and an uneven distribution of solutes [56]. Therefore, we recommend adding an annealing step to the viral formulation during slow freezing to maintain the stability of the formulation (Figure 2).

## 6. Types of Stabilizers

**Figure 3:**
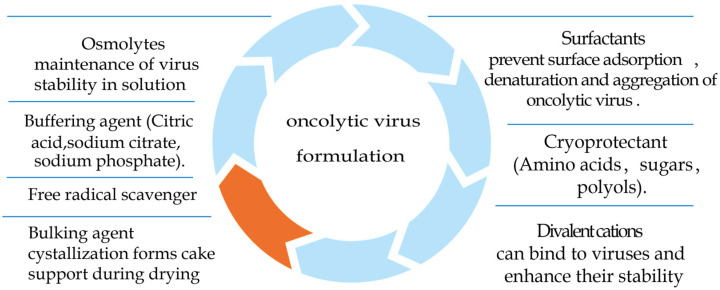
Composition of a typical oncolytic virus formulation. Oncolytic virus formulations contain one more bulking agent than liquid formulations. Orange represents an additional bulking agent for freeze-dried formulations compared to liquid formulations.

Oncolytic virus formulations are usually divided into liquid and lyophilized formulations (Figure 3). In general, lyophilized formulations have greater stability compared to liquid formulations. The cold chain requirements are also relatively low. Freeze-drying is the typical method for maintaining the stability of viral vaccines, and cold chain transport requires only 2~8 °C [61]. However, liquid formulations have relatively lower process requirements than lyophilized formulations because they have fewer lyophilization steps, making them more convenient for early clinical application.

Furthermore, the added drying step may increase the loss of viral activity. Losses of adenovirus potency of ≥0.4 log10 during drying were reported in formulations (35 mM NaCl, 10 mM histidine, 1 mM MgCl_2_, 0.1 mM EDTA, 0.1% *w*/*v* polysorbate 80, 7.5% *w*/*v* sucrose, 0.5% *v*/*v* ethanol, pH 6.6) [62]. Classical liquid formulations include buffers, osmolytes, cryoprotectants, and surface adsorbents. For example, the marketed oncolytic virus formulation T-VEC contains disodium hydrogen phosphate dihydrate (15.4 mg), sodium dihydrogen phosphate dihydrate (2.44 mg), sodium chloride (8.5 mg), inositol (40 mg), and sorbitol (20 mg) [5]. The abandonment of surfactant use in T-VEC formulations may be due to the consideration that the oxidation of commonly used surfactants (PS20 and PS80) may lead to a loss of viral activity. In contrast, the lyophilized formulation adds a bulking agent to the liquid formulation to ensure the quality of the drying step.

### 6.1. Buffers

The first step in formulation design is determining the pH range in which oncolytic viruses are stable. Viruses have different stabilities and solubilities in different pHs. Commonly used buffers for biological formulations are histidine, sodium phosphate, potassium phosphate, citrate, tris, and succinate buffers [63]. The initial pH of the different buffer solutions also varies. The proper buffer solution must be chosen according to the appropriate pH requirement of the virus. It was found that the pH of the buffer solution can change drastically during freezing due to the successive crystallization of the buffer substances. The intensity of the change is related to the type and concentration of the buffer. As the temperature decreases during freezing, the less soluble components of the buffer crystallize preferentially, and the frozen concentrate increases by several orders of magnitude [7]. Two examples of phosphate buffers are sodium phosphate and potassium phosphate. Due to the preferential crystallization of disodium hydrogen phosphate (as dodecahydrate), the order of crystallization is disodium hydrogen phosphate dodecahydrate > sodium chloride dihydrate > potassium chloride, resulting in pH decreases of 3.1 and 2.7 units [64]. In 25 mM and 250 mM succinate buffers, the pH increased by 1–1.2 units upon freezing [65]. Therefore, the crystallization of buffer substances is the root cause of pH drift.

According to the crystallization tendency of buffers, malic acid < citric acid < tartaric acid < histidine glycine ≈ succinate ≈ phosphate [66]. Freezing and low temperatures reduce solubility, making these buffer systems susceptible to crystallization at sub-zero temperatures. There have been several studies on reducing pH changes during freezing (Table 2). Salt co-solutes have been found to improve the pH drift of frozen solutions; in general, salts with large anions such as sulfates increase the acidity of frozen solutions, while halides increase the alkalinity of frozen solutions. The relatively small size of these anions (e.g., halides) means that they usually bind to the ice lattice better than almost any cation. In contrast, relatively large anions (nitrates, acetates) typically do not attach to the ice [67]. For example, Imrichová et al. reported that chloride anions were preferentially incorporated into ice over sodium cations. Ice crystallization was accompanied by increased alkalinity in the frozen concentrated solution [68]. Therefore, we can add salts to regulate the pH changes due to the precipitation or crystallization of buffer components during freezing. In addition, the use of amorphous excipients can also inhibit buffer crystallization such as sucrose, alginate, and cellobiose [64,69,70]. For example, Thorat et al. reported that the addition of cellulose disaccharides attenuated the pH shift of the phosphate buffer on cooling (pH decreased by approximately 1.0 unit), and no buffer salt crystallization was observed [71]. Adding alginate or mannitol as co-solutes to PBS reduced the pH drift on freezing to ~1.7 unit [64]. Finally, reducing the concentration of the buffer substance can also mitigate the pH shift. For example, reducing the concentration of phosphate buffer from 100 mM to 10 mM results in freezing with little change in pH [70].

### 6.2. Penetrant and Cryoprotectant

Osmotic pressure affects the stability of viruses, and higher external osmotic pressure can induce the genome release of viruses [72]. It is known that proteins remain stable in aqueous solutions based on preferential hydration [73]. It is commonly believed that stable co-solutes exhibit a large spatial repulsion. This repulsion effect, in turn, allows for more water molecules on the hydration shell in the bulk solvent. As a result, the hydration shell thickens, allowing proteins to require more energy to unfold [74,75].

On the other hand, osmolytes that cannot preferentially accumulate such as the ionic osmolyte NaCl compromise protein stability by replacing the hydrogen bond between protein and water in direct interactions with the protein fraction [76]. For example, adding NaCl (and other salts) increases the loss of viral titers during F/T [77]. Normally ionic permeants disrupt the hydration shell, and non-ionic permeants such as sucrose, alginate, glycerol, sorbitol, and l-arginine preferentially exclude the hydration shell and help maintain stable virus stereotypes. For example, 1% l-arginine greatly increased the stability of a virus [78]. However, it was found that viral formulations with added divalent cations could also bind to the capsid to consolidate the structure. As demonstrated for other viruses, their removal was shown to destabilize the capsid and promote exfoliation. Caliaro et al. found that prolonged exposure of the viral nucleocapsid to chelating agents or buffers with chelating properties resulted in a 4 °C structural rearrangement, decreasing capsid density. Furthermore, short exposure to 37 °C or freeze–thaw cycles was sufficient to trigger DNA externalization without capsid disassembly. No rearrangements were observed without chelating activity or in the presence of MgCl_2_ or CaCl_2_, suggesting that the depletion of coat-associated divalent cations contributes to exfoliation [50,79]. There is much evidence that adding divalent cations to the formulation increases the stability of a virus [80]. Simultaneous high potassium, but low sodium and low calcium concentrations (simulating the endosomal environment), resulted in viral decidualization. A combination of low sodium, low calcium, and high potassium (20 mM NaCl, 30 mM K+, 0.5 mM MgCl_2_, and 0.2 mM CaCl_2_) in the presence of physiological magnesium concentrations has been reported to trigger the decapitation process [81]. Therefore, formulating viral formulations is recommended to add divalent cations (e.g., MgCl_2_ or CaCl_2_); stabilizers should avoid combinations of low sodium, low calcium, and high potassium and apply ionic permeates, in combination with preferentially accumulating permeates to maintain virus stability in aqueous solutions.

The osmotic agent concentration affects virus stability. Majumder et al. reported that the presence of D sorbitol was effective in reducing nucleocapsid aggregation [82]. However, studies have shown varying degrees of enveloped viral aggregation at permeants greater than 300 mM [83]. Sucrose and PEG are preferentially excluded from proteins at low osmolarity concentrations and preferentially accumulate at higher concentrations [84]; therefore, the concentration must be carefully considered when adding an osmotic agent.

Various excipients are available in solution as osmotic agents and in the frozen state as cryoprotectants, for example, sucrose, sorbitol, and glycerol. During freezing, amorphous substances and proteins are excluded from the ice and crystallization solvent. Sugar can replace water–hydrogen bonds, thus preventing dehydration-induced unfolding so that amorphous substances act as stabilizing agents for proteins in the frozen state [85]. The transition from weakly to strongly associated hydrogen bonds between cryoprotectants and proteins occurs above and below the glass transition temperature [86]. The phenomenon of the final freezing concentration of proteins being proportional to the sucrose concentration [87] also supports the idea that the formation of hydrogen bonds between cryoprotectants and proteins plays a vital protective role.

Commonly used cryoprotectants for viral formulations are disaccharides (alginate and sucrose), polyols (glycerol, mannitol, and sorbitol), gelatin [55], serum albumin, and FBS. The investigators evaluated the cryoprotective function of more than 50 excipients against enveloped viruses. They found that 10% (*w*/*v*) sucrose, sorbitol, or alginate provided the best protection against viruses, with a decrease in infectious titers after three FT cycles at −80 °C [77]. When using 90% (*w*/*v*) alginate:glycerol (1:30), a 0–10% decrease in virus titer was observed after 4 h, while a decrease in more than 70% was observed in the same period when using the control storage buffer formulation [88]. Sorbitol and mannitol may not be suitable cryoprotectants in virus solutions because they tend to crystallize during cooling [89,90]. The dihydrate produced during the freezing of alginate may also crystallize, thus affecting viral stability [89]. Serum albumin and fetal bovine serum have biosafety risks, and it has been found that FBS is not an effective cryoprotectant under refrigeration conditions [89].

Therefore, sucrose may be the preferred cryoprotectant. Previously, it has been found that sucrose in formulations with low protein concentrations induced particle formation. For higher protein concentrations (30 mg/mL and 40 mg/mL), there was a positive effect to hinder particle formation. Sucrose protects the protein from the ice surface and achieves the desired cryoprotective effect only at higher protein concentrations (above 10 mg/mL). There exists an ideal sucrose-to-protein ratio on a U-shaped curve describing the cryoprotective capacity [91].

### 6.3. Surfactant

Surface adsorption on storage containers, ice, and water interface adsorption during freezing and thawing as well as agitation stress during transportation all require the addition of surfactants to solve these problems. Surfactants commonly used in biological formulations such as Pluronic F68, PS20, and PS80 have been shown to greatly benefit viral formulations by reducing surface adsorption [92] (Table 3). However, the formulation of PS80 contained aldehydes, ketones, and hydrogen peroxide, with oxidation preferentially occurring at the double bonds of fatty acid chains [93]. Unlike PS80, PS20 has no unsaturation or sites prone to autoxidation [94]. It has been reported that the amount of hydrogen peroxide in PS80 can increase 1000-fold in under 6 weeks of light conditions. A comparison of the preservation effect of oxidized, unhydrolyzed, and hydrolyzed PS80 on proteins has been carried out. It was found that oxidized PS80 remained protective against protein aggregation and showed increased surface activity [95]. The hydrolysis of polysorbate (PS) can be induced chemically and enzymatically. 

The free fatty acids formed after hydrolysis are hydrophobic and difficult to dissolve in aqueous buffer systems and therefore tend to precipitate and form particles. Hydrolyzed polysorbates lead to a large increase in particle formation during vibrational stress [96] stimulate protein aggregation [97], and result in a slower surface adsorption rate of PS80. Polysorbates 20 and 80 are more stable in acidic hydrolysis than in basic hydrolysis [98]. The effect of the most commonly used buffers on polysorbate degradation has been studied including sodium histidine chloride, sodium citrate, sodium succinate, and sodium phosphate buffers. The degradation rate of polysorbate was highest in the histidine chloride buffer. Therefore, the compatibility between buffers and surfactants is crucial [99]. Protection against oxidative degradation of polysorbate 20 (PS20) and polysorbate 80 (PS80) can be achieved with the addition of butylhydroxytoluene (BHT) and butyl hydroxy anisole (BHA) [100]. PS20 and PS80 solutions containing antioxidants are more stable, exhibiting lower levels of peroxidation, a lower free fatty acid content, a stable pH, an intact polysorbate micelle structure/composition, and less volatile degradation products [100].

Several biologics are now starting to replace polysorbates with Poloxamer 188 [101]. The surfactant Poloxamer 188 is effective in inhibiting viral aggregation. The addition of ≥0.0005% *w*/*v* Poloxamer 188 to viral formulations has been reported to eliminate significant freeze–thaw losses (losses of up to ∼60% in the absence of P188) and reduce rupture rates to ≤1% per F/T cycle [69]. Its use as a parenteral excipient in protein formulations is much less extensive than polysorbate 20 and polysorbate 80 [101]. Poloxamer 188 is slightly more effective than PS20 in preventing protein co-adsorption [102]. PS20 and PS80 have the advantages of being economically available and more comprehensively studied, but they also have significant drawbacks. FM1000 is a series of compounds containing alkyl chains, amino acids, and polyetherimides. The FM1000 family is derived from surfactant scaffolds that are easily structured and synthesized in a simple two-step process. The 14-carbon long-tailed surfactant (14FM1000) is optimal in preventing protein aggregation, and 14FM1000 has the fastest initial adsorption rate [103]. In addition, 14FM1000 is a reversibly adsorbed surfactant, which may enhance its ability to desorb and rapidly adsorb to transient surfaces. The investigators compared the kinetic characteristics of FM1000 at various water/hydrophobic interfaces with polysorbate 20, polysorbate 80, and Poloxamer 188. FM1000 stabilizes the interface by one to two orders of magnitude faster than the other three surfactants, especially when exposed to stirring pressure and new (fresh) interfaces [48]. FM1000 blocked a larger percentage of the interfacial area than PS80. Lower volume FM1000 surface concentrations were sufficient to prevent protein adsorption to the air/water interface, whereas PS80 required increasing concentrations (below the critical micelle concentration, CMC), while fixed FM1000 concentrations (above the relatively low CMC) could also inhibit surface adsorption [104].

### 6.4. Free Radical Scavenger

Chlamydial proteins lose their function when exposed to light due to oxidation by metal ion impurities in raw materials and excipients. Oxidation can be prevented using free amino acids such as methionine and histidine, and metal ion scavengers such as ethanol, EDTA, and DTPA [93]. Free radical oxidation is one of the main deactivation pathways of Ad5 during storage and results in the generation of hydroxyl radicals. Trp, Met, and EDTA are often used in pharmaceutical formulations and provide different oxidative protection [105].

### 6.5. Bulking Agent

Compared to liquid formulations of viruses, lyophilized formulations require the addition of a bulking agent to guarantee the formation of a solid cake when lyophilized. The commonly used bulking agents in lyophilized formulations are mannitol and amino acids. The most studied bulking agent among the amino acids is glycine, whose popularity is due to its high water solubility and eutectic temperature [106]. It shows a higher tendency to crystallize than mannitol. Glycine has been shown to contribute to virus stability as a filler [107]. However, the addition of glycine leads to a lower glass transition temperature. A low glass transition temperature can lead to the severe collapse of the lyophilized formulation at drying temperatures above the Tg. For example, a glycine/sucrose system has been used as a model system, and DSC analysis has shown that the addition of glycine to the sucrose solution led to a decrease in Tg from −32.3 °C to −51.5 °C (a mixture with a sucrose/glycine ratio of 2:5) [108]. Mannitol is another swelling agent commonly used in viral formulations. The high tendency to crystallize in frozen solutions and the high eutectic temperature makes it a good filler. The water content of mannitol in the final lyophilizer affects the lyophilization stability. X-ray powder diffraction, residual water determination, and differential scanning calorimetry were used to analyze the properties of a dried vaccine, and it was found that a low residual water content below 2% made the virus more stable during storage [107]. Time-/temperature-resolved synchrotron X-ray diffraction measurements during freezing and thawing of mannitol/water mixtures have been reported and revealed that mannitol crystallization depends strongly on the cooling rate and is initiated during cooling if the cooling rate is below the critical cooling rate; otherwise, mannitol remains amorphous during freezing [109]. Evidence has shown that no mannitol crystallization was observed when cooling at a cooling rate of 20 °C/min [110]. For 5% mannitol solutions (vial filling volume of 1 to 3 mL; 10 mL glass 53), the critical cooling rate was 15 °C/min, and faster freezing rates resulted in non-crystallization of mannitol [110]. Usually, the annealing step at low temperatures can rescue the behavior of mannitol from not crystallizing when it is frozen rapidly. Not only that, but the addition of an annealing step can also solve the uneven solute distribution formed during freezing. When freeze-drying with sucrose, alginate, or lysozyme as a second solute, mannitol partially crystallizes as MHH (mannitol hemihydrate) and the solute is not uniformly distributed. Annealing the frozen solution at −30 °C for 2 h essentially eliminated it, accompanied by an overall increase in MHH content [111]. However, as with glycine, mannitol may also present the problem of requiring a higher ratio of swelling agent to the stabilizer to ensure their crystallization during the freeze-drying process. For example, a mannitol/sucrose ratio of less than 4/1 will not result in the complete crystallization of mannitol [112]. Although mannitol is the classical filler, Zhang et al., found that mannitol crystallization in the freezing step leads to extensive degradation and loss of potency of adeno-associated virus [112]. However, the researchers did not add a non-surfactant to verify whether the loss of infectivity was due to crystallization. Fillers are known to crystallize, causing a loss of viral activity. Excipients may need to be added to ameliorate the loss of potency due to crystallization. This also highlights the difficulty of studying formulated formulations because of the complexity of the degradation factors involved. There are many types of excipients, and experimental design is difficult. The same excipient under different experimental conditions may yield opposite results. Therefore, we need a more rational experimental design.

## 7. Conclusions

Although studies on the stability behavior of oncolytic viruses are limited, protein and virus stability behaviors are similar enough to draw on protein formulations to reduce the probability of ineffective formulations, and the economic and time costs of trial and error. Nevertheless, viral stability has unique characteristics. From the above discussion, it is clear that different species of oncolytic viruses have different optimal pH values and cannot be generalized. In the past, when studying the optimal pH values, the infectivity of viruses has been verified under different conditions only from the point of view of physical stability without adding the actual infection efficiency, which may lead to obtaining incorrect results. Divalent cations can bind to viruses and increase their stability [50,79], but the mechanism of their stabilization is not fully understood. There are also some common mechanisms that still need to be investigated. It is known that the freezing process leads to inhomogeneity of the concentration in the frozen layer due to the freezing of water. The addition of an annealing step results in homogeneous proteins and unshaped material [56]. Does this indicate that unfrozen viruses and solutes are still circulating with each other after freezing? The potential mechanism by which adding sucrose or reducing the buffer concentration could reduce pH drift [70] deserves to be investigated. It is relevant to circumvent the issue of degradation when using polysorbates as surfactants and the need to circumvent toxicity issues when using new surfactants. It would be interesting to investigate whether containers with surface activity can solve the problems of degradation and toxicity.

The interaction of excipients with tumor viruses may lead to a loss of activity. The variety of excipients should be minimized, and the identification of multifunctional excipients and the concentration of excipients should be the direction of research. On the other hand, the identification of new stabilizers could be a potential avenue, although this approach would be associated with toxicity and regulatory barriers related to the approval process [53]. The selection of suitable excipients should provide stabilization of tumor viruses. Although the stabilization mechanism is not fully understood, it contains a variety of compounds, including buffers, divalent cationic salts, and sugars that act as both permeation and cryoprotective agents, surfactants, and antioxidants. Since excipients can affect the physical properties of oncolytic viruses and vice versa, formulation composition should be based on sound experiments and appropriate stability studies. 

## Figures and Tables

**Table 1:** The physical stability of different species of oncolytic viruses at different pH levels.

Virus Type	Aggregation of Viruses	Nucleocapsid Transition Temperature	Nucleocapsid Dissociation	Referencs
Adenovirus type 5	Virus aggregates extensively at pH 4–5 and to a lesser extent at pH 6	The transition temperature increases as pH 8–4 decreases	The lowered pH (pH 5) destabilizes the Ad capsid and causes protein dissociation from the capsid apex	[9,23]
Adenovirus type 2	Extensive virus aggregation at pH 3–4 and rapid loss of virus activity at pH5	The transition temperature increases as pH 8–5 decreases	Weak acidic pH induces amphiphilicity of adenovirus capsid proteins and may help Ad2 escape from acidic endocytic vesicles	[31,32]
Adenovirus type 4		The transition temperature increases as pH 8–4 decreases		[33]
Herpes simplex virus	Aggregation tendency increases at pH 5.5–6	The transition temperature increases as pH 8–5.5 decreases	(pH 5–6) Low pH-triggered conformational changes of gB are reversible, although irreversible low-pH inactivation	[19,34]
Measles virus	Widespread aggregation of pH 4–5 viruses	The overall transition temperature decreases as pH 8–4 decreases, Tm is 50 °C at pH 6 and 7, Tm is 47 at pH 8.		[25]
Coxsackie virus		More stable in acidic and neutral conditions than in alkaline conditions	Does not trigger stripping shells in both acidic and neutral environments	[29,35]
Newcastle disease virus			Promotion of viral matrix proteolysis under acidic conditions at pH 4	[26]

**Table 2:** Example of mitigation of pH drift during freezing.

Buffer (Concentration)	Excipients	Storage Conditions	Process	Reference
50 mM sodium phosphate solution and potassium phosphate solution	Ionic cryoprotectants (e.g. TMACl)	77 K	With the addition of 0.1M TMACl, the pH of the sodium phosphate solution decreased from 7.5 to 7.1 after freezing, and the pH of the potassium phosphate solution remained unchanged.	[67]
Disodium hydrogen phosphate-potassium dihydrogen phosphate buffer	Sucrose	−20 °C	The pH decreased from 7.4 to 4.3 when sucrose was not added, and only decreased by 1.1 when sucrose was added.	[69]
Sodium phosphate buffer		−20 °C	Buffer pH is 7.2, concentration less than 14mM, pH only about 1 pH lower	[69]
Phosphate buffer solution (PBS)	Seaweed sugar or mannitol	−20 °C	The pH decreased significantly (~4.3 units) when the PBS solution was frozen. The addition of as co-solvent to PBS reduced the magnitude of pH drift to ~1.7 units.	[64]
25 mM succinic acid buffer	Sucrose	−20 °C	The increase in pH from 5.0 to 6.2 during freezing was attenuated by the addition of only 2% sucrose, while higher concentrations (4% and 8% sucrose) had a better inhibitory effect on the pH change.	[65]
100 mM phosphate buffer solution	Fibrous disaccharides	−25 °C	The addition of fibrous disaccharides attenuated the pH drift on cooling (~1.0 unit decrease in pH), and no evidence of buffer salt crystallization or protein aggregation was observed. Decreasing the buffer concentration to 10 mM also maintained pH stability	[70]

**Table 3:** Advantages and disadvantages of different types of surfactants.

Surface Adsorbent	Advantages	Disadvantages
Polysorbate 20	Nonionic surfactants are superior to ionic surfactants and have better anti-polymerization effects Polysorbate 20 does not contain any unsaturation or sites prone to autoxidation. It is economically accessible and more comprehensively studied.	Slowest adsorption rate to surfaces compared to the other three surfactants Forms faster particle formation for formulations containing PS20 compared to those containing PS80 Release of free fatty acids by enzymatic hydrolysis of PS20
Polysorbate 80	Economical and easy to obtain with comprehensive research. Anti-adsorption ability is better than PS20	The PS80 formulation was shown to contain aldehydes, ketones, hydrogen peroxide and ROOH, with oxidation preferentially occurring at the double bonds of fatty acid chains, which predisposes to autoxidation and peroxide formation. Hydrolysis to form particles
Poloxamer 188	P188 is slightly more effective than PS20 in preventing co-adsorption	A number of biologics are now beginning to replace polysorbate with Poloxamer 188, but there is still little research on Poloxamer 188. Its use as a parenteral excipient in protein formulations is much less extensive than that of polysorbate 20 and polysorbate 80
FM1000	Easy to structure and simple synthesis steps. Its stabilization of the interface is 1–2 orders of magnitude faster than the other three surfactants, especially when exposed to stirring pressure and new interfaces FM1000 blocks a larger percentage of the interfacial area than PS80 The lower volume FM1000 surface concentration is sufficient to prevent protein adsorption to the Fewer protein particles are formed in the presence of FM1000. To achieve the same surface adsorption capacity PS concentration has to be higher	Compared to polysorbate 20 and polysorbate 80, its use as a parenteral excipient in protein formulations is much less extensive

## Data Availability

Not applicable.
